# Numerical Modeling and Simulation of Non-Invasive Acupuncture Therapy Utilizing Near-Infrared Light-Emitting Diode

**DOI:** 10.3390/bioengineering10070837

**Published:** 2023-07-15

**Authors:** Sundeep Singh, Andres Escobar, Zexi Wang, Zhiyi Zhang, Chundra Ramful, Chang-Qing Xu

**Affiliations:** 1 Faculty of Sustainable Design Engineering, University of Prince Edward Island, Charlottetown, PE C1A 4P3, Canada; 2 Department of Biomedical Engineering, McMaster University, Hamilton, ON L8S 4L8, Canada; escoba3@mcmaster.ca; 3 Department of Engineering Physics, McMaster University, Hamilton, ON L8S 4L8, Canada; wangz418@mcmaster.ca; 4 Advanced Electronics and Photonics Research Center, National Research Council Canada, Ottawa, ON K1A 0R6, Canada; zhiyi.zhang@nrc-cnrc.gc.ca (Z.Z.); chundra.ramful@nrc-cnrc.gc.ca (C.R.)

**Keywords:** bio-heat transfer, light-tissue interaction, photothermal effect, acupuncture, acupoints, thermal therapy, computational modeling

## Abstract

Acupuncture is one of the most extensively used complementary and alternative medicine therapies worldwide. In this study, we explore the use of near-infrared light-emitting diodes (LEDs) to provide acupuncture-like physical stimulus to the skin tissue, but in a completely non-invasive way. A computational modeling framework has been developed to investigate the light-tissue interaction within a three-dimensional multi-layer model of skin tissue. Finite element-based analysis has been conducted, to obtain the spatiotemporal temperature distribution within the skin tissue, by solving Pennes’ bioheat transfer equation, coupled with the Beer-Lambert law. The irradiation profile of the LED has been experimentally characterized and imposed in the numerical model. The experimental validation of the developed model has been conducted through comparing the numerical model predictions with those obtained experimentally on the agar phantom. The effects of the LED power, treatment duration, LED distance from the skin surface, and usage of multiple LEDs on the temperature distribution attained within the skin tissue have been systematically investigated, highlighting the safe operating power of the selected LEDs. The presented information about the spatiotemporal temperature distribution, and critical factors affecting it, would assist in better optimizing the desired thermal dosage, thereby enabling a safe and effective LED-based photothermal therapy.

## 1. Introduction

Over the past two decades, there has been a significant increase in the use of complementary and alternative medicine worldwide, across all age groups [1,2,3,4,5,6,7]. In particular, complementary and alternative medicine includes diverse and abundant traditional practices, such as naturopathy, chiropractic care, acupuncture, Ayurveda, and homeopathy. Among these therapies, acupuncture has received significant attention, and is one of North America’s fastest-growing complementary and alternative medicine therapies [8]. Acupuncture is a nonpharmacological and minimally invasive traditional Chinese medicine therapy that originated more than 2000 years ago, and has gradually spread and become quite popular in the West [9,10]. Significant research efforts have been devoted in the past four decades to investigating acupuncture’s clinical effectiveness and mechanisms of action, with more than 10,000 randomized controlled trials published since 1975 [10,11,12,13]. However, the exact explanation for the biological mechanism of action involved in acupuncture still remains elusive, which has hindered its modern development and integration into conventional medicine [14].

Today, acupuncture is widely used to treat a plethora of diseases, such as musculoskeletal pain, gastrointestinal disorders, stroke, gynecological diseases, neurological disorders, and various types of incurable chronic disease [1,15,16,17,18]. During modern acupuncture therapy, very thin needles are inserted into the skin at certain specific acupoints in the human body. The acupuncture needles are then stimulated by applying mechanical, electrical, or other physical forms of energy, to activate various nerve receptors directly or indirectly [17,19]. Several hypotheses have been proposed to describe the molecular pathways associated with the therapeutic effects of acupoint stimulations. One such recent hypothesis is related to the activation of mast cells during acupuncture, which leads to analgesic effects [14]. Moreover, the distinction between acupoints and non-acupoints is made based on the concentration of mast cells. As such, acupoints have a higher concentration of mast cells than any other location. The stimulation of the acupoints during acupuncture therapy results in the degranulation of mast cells, which further modulates neighboring cell behaviors by releasing biological substances, thereby enhancing vascular permeability and nerve activation [14].

In the broader context of acupuncture therapy, the stimulation of the acupoints can be performed through mechanical (acupuncture), heat (moxibustion), and electrical (electroacupuncture) treatments. The present study focuses on the stimulation of acupoints with heat, also called moxibustion therapy. As such, moxibustion is a non-invasive thermal therapy that completely omits the need for needle insertion, thereby mitigating any chance of pain, infection, or damage to the skin layer. Moxibustion is a relatively simple and tolerable treatment, whereby dried mugwort (moxa) is burned over the acupoint, either directly touching the skin or at a certain distance (or gap) from the skin surface [20,21]. Existing research suggests that the high temperature (approximately 500 °C) of burning moxa sticks could result in a temperature rise within the skin tissue ranging between 38 °C and 50 °C [22,23,24]. However, the safe and effective temperature to avoid the patient’s discomfort and irreversible damage has been reported to be above 42 °C and below 50 °C [24,25]. Furthermore, moxibustion is typically performed for 10–30 min, repeated several times in a week for a few weeks, and is able to provide a thermal stimulus to the depth of 2–4 mm, affecting both the shallow and deep skin tissue [22,23]. The therapeutic effects induced during moxibustion are related to a combination of heat effects, nonthermal radiation effects, and pharmacological effects [22,24,26,27]. Among these, heat stimulation is believed to be one of the critical factors directly related to the efficacy of moxibustion therapy.

Furthermore, the major disadvantage of moxibustion is related to producing harmful gases and odors by burning moxa sticks, similar to tobacco smoke and air pollutants [23,25]. Several alternatives have been explored to address this disadvantage, and to attain similar therapeutic effects related to thermal stimulation during moxibustion therapy but without the actual production of smoke or odor. More recently, the applications of lasers and ultrasonic devices have also been explored to replace moxa sticks and attain a similar rise in temperature within the deep tissues to that achieved in moxibustion therapy [23,25,28,29]. Moving in this direction, in the present study, we aim to propose and evaluate the feasibility of moxibustion-like treatment utilizing near-infrared LED to provide thermal stimulus to the tissue in a completely non-invasive fashion.

Compared to the lasers typically used in complicated clinical settings, LEDs are safer and more cost-effective, and are well-tolerated by patients of all age groups. Furthermore, there are a plethora of visible blue and red light wavelength LED-based devices already available on the market, used by either patients or healthy individuals, independently in their homes, for some generic dermatological applications [30,31,32]. However, to the best of the authors’ knowledge, no studies (or devices) are available in the literature that explore the feasibility of near-infrared LEDs as a reasonable alternative to moxa sticks during moxibustion therapy, and that quantify the relationship between the light-tissue interactions during such a treatment. The selection of near-infrared LEDs is also based on the fact that it reaches the maximum depth in the skin. In the following sections, we first characterize the light irradiation of selected LEDs on the surface of the tissue. Then, we utilize a numerical modeling framework to quantify the temperature rise within the skin tissue subjected to the Gaussian irradiation profile of the LED. Furthermore, parametric studies have been conducted, to investigate the effect of the applied power, irradiation duration, and LED distance from the skin surface on the temperature rise attained at different depths in the skin tissue. The results of the parametric analysis reported in the present study will be helpful in better understanding the effects of various extrinsic factors on the LED-based heating of skin tissue. They would pave the way to developing cost-effective and easy-to-use LED-based wearable devices, to provide on-demand physical stimulus at home, without needing to go to a clinic.

## 2. Materials and Methods

This section provides details of the computational domain and numerical framework adopted to study the effect of LED irradiation on heat transfer in skin tissue. The optical and thermal properties of the tissue, governing equations, and boundary conditions are also presented. Finally, the details of the characterization of the irradiance profile for the selected LEDs, and the experimental setup utilized to test and validate the developed numerical model, have been provided.

### 2.1. Finite-Element-Method (FEM)-Based Model

**Figure 1:**
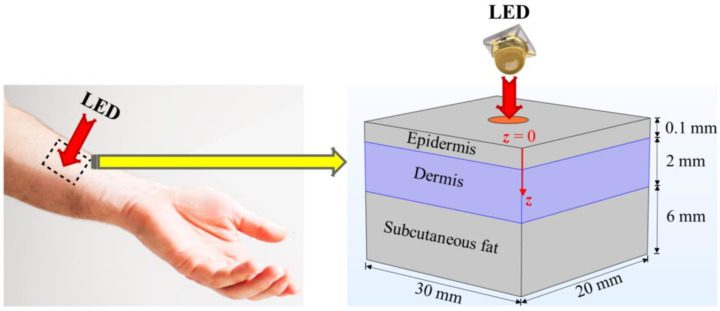
Schematic of the 3D model of the three-layered skin considered in the present study.

Figure 1 presents the computational domain of skin tissue considered in the present study. The skin model comprises three layers: the epidermis, the dermis, and subcutaneous tissue [33,34,35]. Each layer has distinct optical and thermal properties. LEDs were placed on the top surface of the skin tissue, with variable distances between the top of the LED surface and the epidermis skin layer. The main motive of the present study was to quantify the interaction between the LED irradiance and the temperature rise within the skin tissue. In addition, heat transfer analysis within biological tissue is highly complex, due to the heterogeneous vascular structure, blood flow in the complex network of arteries and veins, metabolic heat generation, and dependence of the tissue properties on the physiological conditions [36,37,38]. Pennes’ Fourier-law-based bioheat transfer model is widely used for predicting the temperature distribution within the human body for numerous biological and medical applications, due to its simplicity, computational efficiency, and cost-effectiveness. Notably, Pennes’ model is a continuum model, whereby the blood vessels are not individually incorporated into the computational domain; instead, their effect is lumped into a single factor known as the “blood perfusion rate”.

The generalized version of Pennes’ bioheat transfer equation utilized in the present study for evaluating the LED-induced heating is represented as [38]:

(1) $$\rho c_{p}\frac{\partial T}{\partial t}=\nabla \cdot \left(k\nabla T\right)-\rho_{b}c_{b}\omega_{b}\left(T-T_{b}\right)+Q_{met}+Q_{source}$$

where *ρ* is the density of the tissue (kg/m^3^); *c_p_* is the specific heat capacity of the tissue (J/kg/K); *k* is the thermal conductivity of the tissue (W/m/K); *T* is the temperature of the tissue that will be quantified from Equation (1) (K); *ρ_b_* is the density of the blood (=1050 kg/m^3^) [39]; *c_b_* is the specific heat of the blood (=3617 J/kg/K) [39]; *ω_b_* is the blood perfusion rate (s^−1^); *T_b_* is the arterial blood temperature (=310 K); *Q_met_* is the metabolic heat generation (=368 W/m^3^) [34,35]; and *Q_source_* represents the energy absorption of the LED irradiation (W/m^3^).

The LED heat source has been modeled using the Beer-Lambert law in the numerical analysis, which is represented as [34]:

(2) $$Q_{source}=\mu_{eff}\cdot I\cdot e^{-\mu_{eff\cdot }z}$$

where *µ_eff_* is the effective attenuation coefficient (m^−1^); *z* is the depth of the tissue (m); and *I* is the LED irradiation intensity (W/m^2^) given by [40]:

(3) $$I(x,y)=I_{o}\cdot e^{-\;\;\frac{\left(x^{2}+y^{2}\right)}{2\sigma^{2}}}=\frac{P}{2\pi \sigma^{2}}\cdot e^{-\;\;\frac{\left(x^{2}+y^{2}\right)}{2\sigma^{2}}}$$

where *I_0_* is the maximum irradiation intensity at the center of the 2D Gaussian profile of the LED beam (W/m^2^); *σ* is the standard deviation of the LED beam Gaussian profile; and *P* is the LED radiometric power (W). As mentioned, the LED operates at a near-infrared wavelength of 850 nm. Furthermore, the effective attenuation coefficient that accounts for the absorption and scattering phenomena of LED light in skin tissue, based on diffusion approximation, is given as [34]:

(4) $$\mu_{eff}=\sqrt{3\mu_{a}(\mu_{a}+{\mu^{\prime }}_{s})}$$

where *µ_a_* is the absorption coefficient of tissue (m^−1^), and *µ′_s_* is the reduced scattering coefficient of the tissue (m^−1^), both approximated at a wavelength of 850 nm for different skin layers, from [41]. The thermal and optical properties of the different skin layers (the epidermis, dermis, and subcutaneous fat) considered in the numerical simulations are summarized in Table 1 [34,35]. More details of the characterization of material properties utilizing photothermoacoustic spectroscopy can be found in [42].

FEM-based COMSOL Multiphysics 6 (COMSOL, Inc., Burlington, MA, USA) software was adopted to solve the spatiotemporal temperature distribution obtained within the skin tissue subjected to LED irradiation. The computational domain was discretized using unstructured tetrahedral mesh elements in COMSOL. A grid independence solution was obtained by refining the mesh until the error associated with the maximum temperature prediction was less than 0.5% between two contiguous meshes. The optimal number of total mesh elements considered in the present study was 162,118. The initial temperature of the skin tissue was assumed to be the same as the core body temperature of the human body, 37 °C. The Gaussian profile obtained from experimental characterization was applied at the top surface of the computational domain of the skin, at the center, for 10 min. All the computational domain boundaries, except for the top, were applied with thermal insulated boundary conditions. The top surface of the computational domain was subjected to the natural convection and radiation boundary conditions given by $\left(-k\nabla T=h(T-T_{\infty })\right)$, and $\left(-k\nabla T=\varepsilon \sigma (T^{4}-T_{\infty }^{4})\right)$, respectively, where *h* is the convective heat transfer coefficient assumed to be 10 W/m^2^ K [23,35]; *T*_∞_ is the surrounding ambient temperature, considered to be 25 °C [23,35]; *ε* is the emissivity of the skin surface, assumed to be 0.95 [23,34,35]; and *σ* is the Stefan-Boltzmann constant.

### 2.2. LED Irradiance Profile

**Figure 2:**
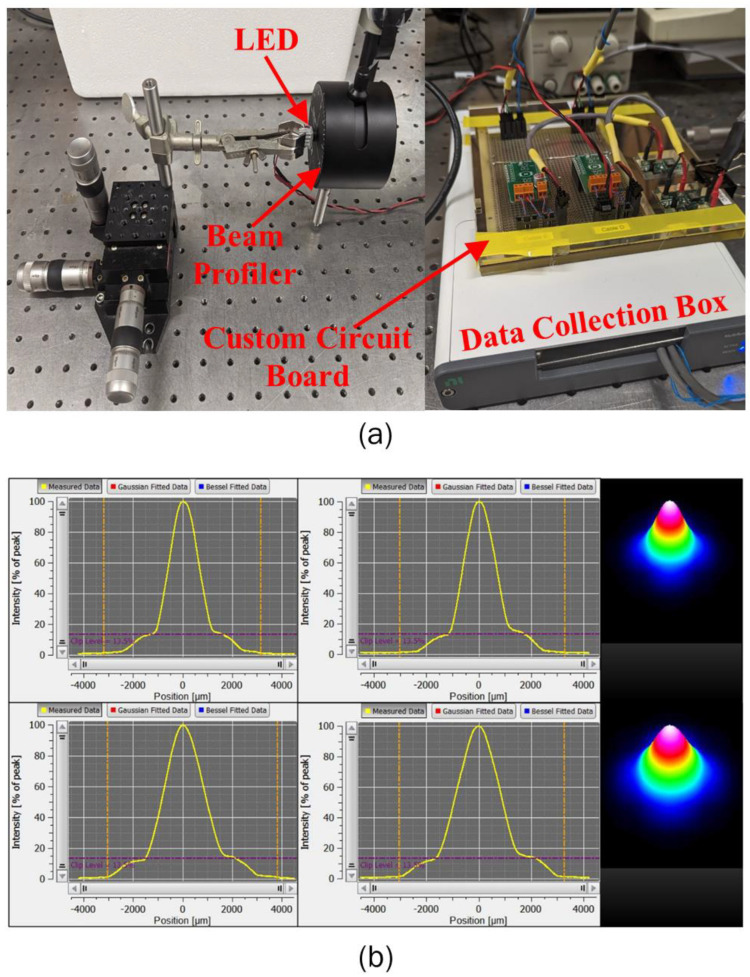
(**a**) The experimental setup used to determine the LED beam profiles. The different components of the experimental setup are the XYZ stage, LED heatsink, beam profiler, and a custom-made circuit board for operating the LEDs. (**b**) A snapshot of a typical beam-profiling measurement. From left to right: the 2D *x*-axis, 2D *y*-axis, and 3D beam profiles of the 90° (top row) and 150° LEDs (bottom row).

The characterization of the optical properties of the selected near-infrared LEDs (LUXEON IR Domed Line, Lumileds Holding B.V.) operating at a wavelength of 850 nm, with 90° and 150° emission patterns representing full-width half maximum (FWHM) angles, was performed utilizing a Thorlabs BP209IR1 beam profiler, as shown in Figure 2a. The XYZ stage was utilized to make micron-level adjustments that would facilitate the alignment of the LED with the center of the beam profiler, to ensure a properly characterized beam spread. This XYZ stage was fitted with a rod that allowed a clamp holding the LED-heat-sink combination to move freely. Once aligned, the LED was moved as close to the aperture as possible, and its position was recorded on the XYZ stage as a relative position zero. Having the LED measured physically close to the aperture would help increase the accuracy of the beam profile shape, its peak intensity, and other optical features, as the entirety of the light produced would be captured within the aperture. With this setup, we could then take beam profile measurements at different relative distances, to better characterize the beam shape and how it would change at increasing distances, to be used as the input in our developed numerical models for predicting the spatiotemporal temperature distribution within the biological tissue. Before any measurements were taken, the beam profile was set to accept the LED operating wavelength of 850 nm, as well as being set to operate at the maximum aperture width of 9.0 mm, and at a scanning rate of 20 Hz. With the increased scanning rate, the resolution of our profiles was increased, and helped to reduce the warping effect of the potential saturation incurred by the LED on the beam profiler. Customized software and a customized circuit board were used in the experiments. The LED profile for both LEDs was represented by a Gaussian-fit curve, as represented in Figure 2b. This Gaussian irradiation profile for both LEDs was obtained for different operating currents and voltages, and thus different operating power. The obtained Gaussian irradiance profile was provided as an input at the top of the skin surface in the numerical model for quantifying the light-tissue interaction. The obtained data were in agreement with the manufacturer’s specifications and reported values.

### 2.3. Experimental Setup for Testing the LEDs

**Figure 3:**
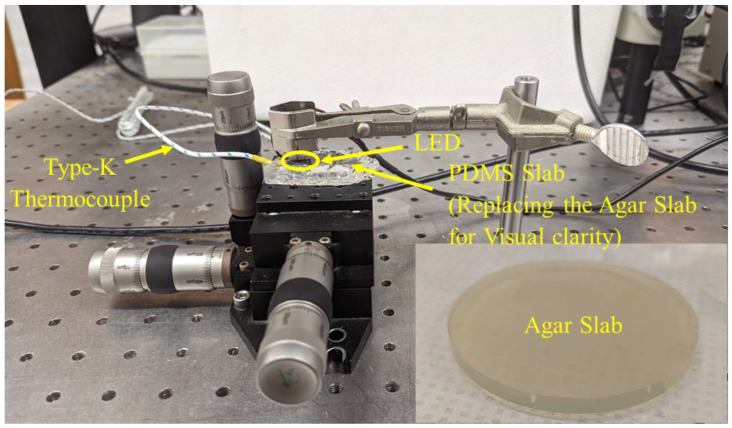
Photographic view of the experimental setup for measuring the temperature profile. The snapshot in the bottom right-hand corner is a sample of the actual agar gel used in the temperature profile experiments.

Agar gel was prepared with a 2.6% (*w*/*v*) concentration of agar powder mixed in DI water. The recipe for the agar gel was adopted from [43], owing to the availability of optical and thermal properties at that concentration. The fabricated agar gel was cylindrical, with a height of 0.9 cm and a diameter of 7.5 cm. Figure 3 presents the experimental setup utilized for testing the LEDs on the agar gel. The LED was applied at the top surface of the agar gel, and a type-K thermocouple was gently inserted in the agar gel, to keep the monitoring tip at a distance of 2 mm just below the center of the LED from the top surface. For experimental validation, three sets of trials (for each LED) were conducted, to quantify the temperature profile at a depth of 2 mm within the agar tissue exposed to the 90° and 150° LEDs operated at a current of 0.802 A and a voltage of 3.6 V, for 5 min.

## 3. Results

### 3.1. Experimental Validation

**Figure 4:**
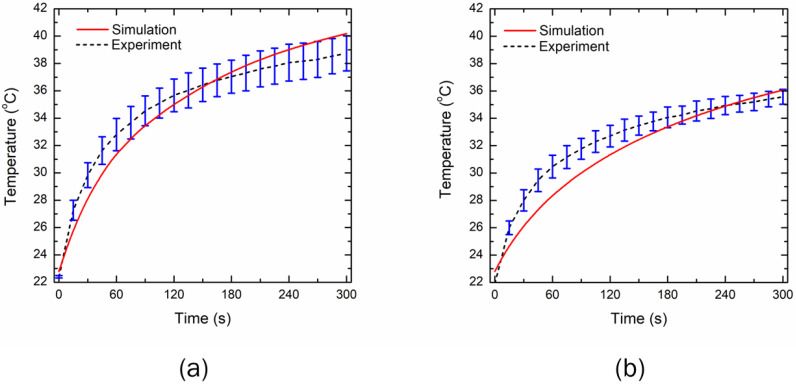
Comparison of the experimentally measured and numerically predicted temperature profiles at a depth of 2 mm for LEDs with emission patterns of: (**a**) 90°, and (**b**) 150°.

The developed model’s fidelity and integrity have been evaluated by comparing the results predicted from the numerical simulation with those obtained numerically from experimental studies on agar phantom under the same environmental conditions. Notably, the temperature profile obtained at the location of 2 mm away from the center of the LED along the tissue depth was used for validation purposes. Motivated by [43], the thermal and optical properties of the agar phantom were considered to be *ρ* = 1050 kg/m^3^, *c_p_* = 4219 J/kg/K, *k* = 0.66 W/m/K, *µ_a_* = 40 m^−1^, and *µ′_s_* = 530 m^−1^. The effects of blood perfusion and metabolic heat generation were neglected in the numerical model, to have consistency with the agar phantom. The comparison of the temperature profile attained with 5 min of LED irradiation has been presented in Figure 4. The mean standard deviation along the whole trend of the experimental tests is 3.19% and 2.1%, for the 90° and 150° emission pattern LEDs, respectively, thus proving a limited variability across the experiments. The correlation factor, R, between the numerically predicted and experimental data after 300 s of LED irradiation is found to be 0.98 and 0.97, for the 90° and 150° emission pattern LEDs, respectively. The mean absolute error is found to be 0.95 °C and 1 °C, for the 90° and 150° emission pattern LEDs, respectively, which shows a good performance. As shown in Figure 4, there is a good agreement between the temperature profile obtained with the numerical simulation and with experiments, for both the 90° and 150° emission pattern LEDs, thereby lending great confidence to the results reported and discussed in the following sections. Next, we will present the results of the numerical model, extended to incorporate the optical and thermal properties of skin tissue, including microvascular blood perfusion.

### 3.2. Effect of Input LED Power on Temperature Rise within the Skin

**Figure 5:**
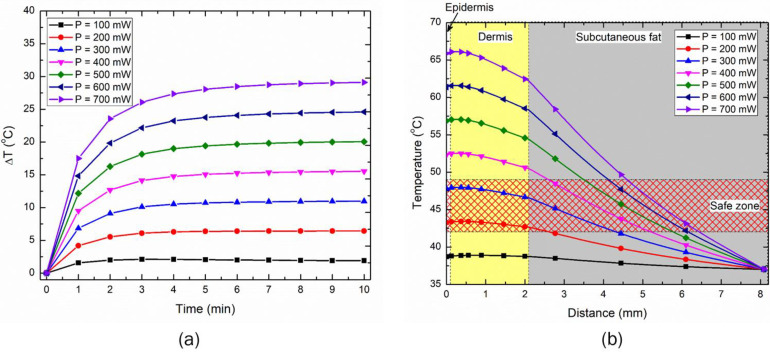
The effect of an input power of 90° emission pattern LED on (**a**) the maximum temperature rise with time within the skin tissue, and (**b**) the temperature variation along the depth of the skin after 10 min of LED irradiation.

In this section, we will report the results of the parametric analysis conducted to quantify the effect of the input LED power and the duration on the temperature rise attained within the skin tissue. The treatment time for the therapy was selected to be 10 min to attain thermal stimulus (>43 °C) at the depth ≥ 2 mm, consistent with typical moxibustion therapy. The parametric analysis was conducted on the two LEDs operating at a near-infrared wavelength of 850 nm, with 90° and 150° emission patterns representing full-width half maximum (FWHM) angles. Although these LEDs can be operated at the maximum power of 1000 mW, to avoid any heating and self-damaging issues, the maximum operating power of the LED was kept as 700 mW. Figure 5a presents the transient temperature increase obtained within the skin tissue when the 90° LED was placed at the top of the three-layer skin model, with direct contact. As evident from Figure 5a, the increase in the operating power of the LED resulted in a corresponding increase in the maximum temperature rise within the skin tissue. The relative jump in maximum temperature attained by increasing the LED power by a factor of 100 mW was approximately the same for all the cases. Further, the maximum temperature reached within the skin tissue was less than 50 °C for the LED operating at 300 mW, beyond which the maximum temperature was greater than 50 °C. For all the LED operating powers, initially, the temperature rose steeply and later stabilized and saturated with time. The higher the LED’s operating power, the longer the time required to attain this saturation. For all considered cases, the saturation was attained within 5–6 min of LED therapy. This highlights that the maximum temperature will be constant and will not increase with an increase in treatment time, i.e., the maximum temperature attained after 10 min will be the same after 15 min or 30 min, or even after one hour of LED therapy. However, the zone of the skin tissue experiencing thermal effects may still enhance with time, due to the conduction of heat deposited within the tissue.

Figure 5b presents the temperature distribution attained after 10 min of LED therapy within the skin tissue, as a function of the depth from the skin’s outermost layer and from the LED’s center. As evident from Figure 5b, the maximum temperature was not attained at the outer epidermis layer of skin; indeed, it occurred at the dermis layer. This can be attributed to the natural convection cooling effects prevalent at the outer epidermis layer, lowering the temperature slightly. After attaining the peak, the temperature continuously declined with the depth of skin tissue. Furthermore, the increased operating power of the LED increased both the temperature and the depth to which the higher-temperature effects were felt within the skin tissue. Notably, previous physiological studies have highlighted the activation of cutaneous thermosensitive receptors (TRPV channels) with increased tissue temperature, inducing different biological and chemical reactions in the human body [22,23,26,27,44]. For example, TRPV1 and TRPV2 can be activated when the heated tissue temperature exceeds 43 °C and 53 °C, respectively. Moreover, the rise in temperature at 44.5–46.5 °C can activate A-fibre mechano-heat-sensitive nociceptors in the human skin [45]. The cholesterol-lowering effect is far more pronounced at 46 °C than at 38 °C [46]. Moreover, attaining a temperature above 50 °C will result in thermal ablation, i.e., inducing irreversible damage within the skin tissue. Thus, the safe working limit of the proposed LED-based photothermal therapy would be in the temperature range of 43–48 °C, similar to the safe and effective range of moxibustion therapy. Based on the parametric studies conducted on the 90° LED, the safe operating power resulting in therapeutic effects has been found to be in the range of 200–300 mW, as highlighted in Figure 5b.

**Figure 6:**
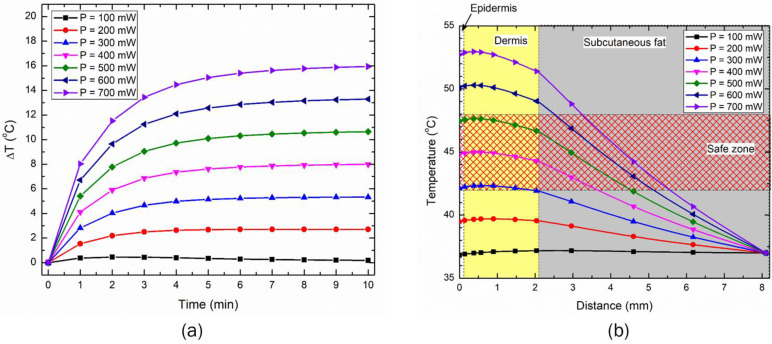
The effect of an input power of 150° emission pattern LED on (**a**) the maximum temperature rise with time within the skin tissue, and (**b**) the temperature variation along the depth of the skin after 10 min of LED irradiation.

The effect of operating power of 150° LED on the maximum temperature rise has been presented in Figure 6a. Again, as is evident, the increase in the operating power of the LED resulted in an increase in the maximum temperature rise within the skin tissue, which was somewhat proportionate to the increase in power. The temperature distribution obtained within the skin tissue after 10 min of LED therapy is presented in Figure 6b. The safe operating range of power to obtain therapeutic effects with 150° LED-based photothermal therapy has been found to be 300–500 mW, as shown in Figure 6b.

**Figure 7:**
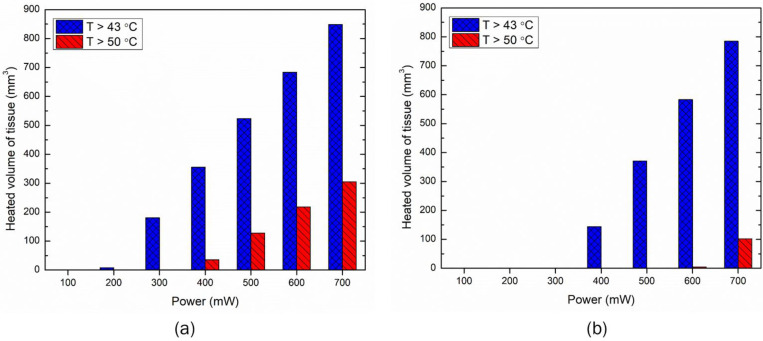
The variation in the heated volume obtained using temperatures greater than the 43 °C and 50 °C protocol after 10 min of LED irradiation with emission patterns of (**a**) 90°, and (**b**) 150°.

Furthermore, Figure 7 presents the heated volume of the skin tissue after 10 min of LED irradiation, quantified with the isotherms of 43 °C and 50 °C. The goal of the proposed LED therapy is to maximize tissue volume attaining a temperature greater than 43 °C, and mitigate any chances of reaching 50 °C anywhere within the skin tissue. The heated volume obtained from the parametric analysis, as presented for the 90° (in Figure 7a) and 150° (in Figure 7b) emission patterns, will assist in selecting the safe operating power range, to avoid the temperature that initiates the ablation of the tissue. As depicted in Figure 7, the heated volume abruptly increased with the increased operating power of the LED. The initiation of the ablation zone (T > 50 °C) occurred at the power of 400 mW and 600 mW, for the LEDs with emission patterns of 90° and 150°, respectively. This highlights that the safe operating power for 90° and 150° should be less than 400 mW and 600 mW, respectively, to avoid the occurrence of thermal ablation leading to irreversible damage to the skin tissue.

**Figure 8:**
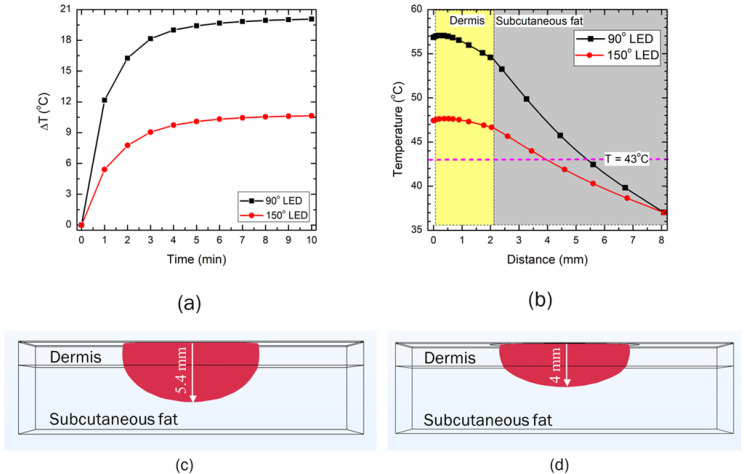
The effect of the LED emission patterns of 90° and 150° operating at an input power of 500 mW on (**a**) the maximum temperature rise within the skin tissue, and (**b**) the temperature distribution along the depth of the skin tissue. Heating zone obtained with the isotherm of 43 °C after 10 min of LED irradiation with the emission pattern of (**c**) 90°, and (**d**) 150°.

Figure 8 directly compares the two selected LEDs, with emission patterns of 90° and 150°, operating at a power of 500 mW. As evident from Figure 8a, the maximum temperature rise attained within the skin tissue is significantly higher for the 90° LED than for the 150° LED. The temperature distribution obtained within the skin tissue after 10 min of LED therapy is presented in Figure 8b, again highlighting the performance of the 90° LED being far superior to that of the 150° LED. This can be attributed to the fact that the emission angle of the 90° LED was more focused and thus could deposit a higher amount of energy in a focused area, compared to the 150° LED that had a higher divergence angle and, thus the light intensity could not penetrate to greater depths. The standard deviation of the Gaussian profile (σ) for the 90° and 150° LEDs was found to be 4.6 mm and 7 mm, respectively, highlighting the irradiation area of the 150° LED being higher, compared to the 90° LED. Therefore, if the goal is to deliver the heat to a greater depth, one would use a 90° LED, and if the goal is to heat up a large area at a lower penetration depth, one would use a 150° LED. The isotherms of 43 °C obtained after 10 min of LED-based photothermal therapy are presented in Figure 8c. As depicted in this figure, the depth to which the effect of the 43 °C temperature is felt is 5.4 mm for the 90° LED, compared to 4 mm for the 150° LED. Thus, the selection of the LED based on both the operating power and emission angle basis would significantly affect the spatiotemporal temperature distribution, and hence the efficacy of LED-based therapy.

### 3.3. Effect of Distance between the LED and Skin Surface on Heat Transport

**Figure 9:**
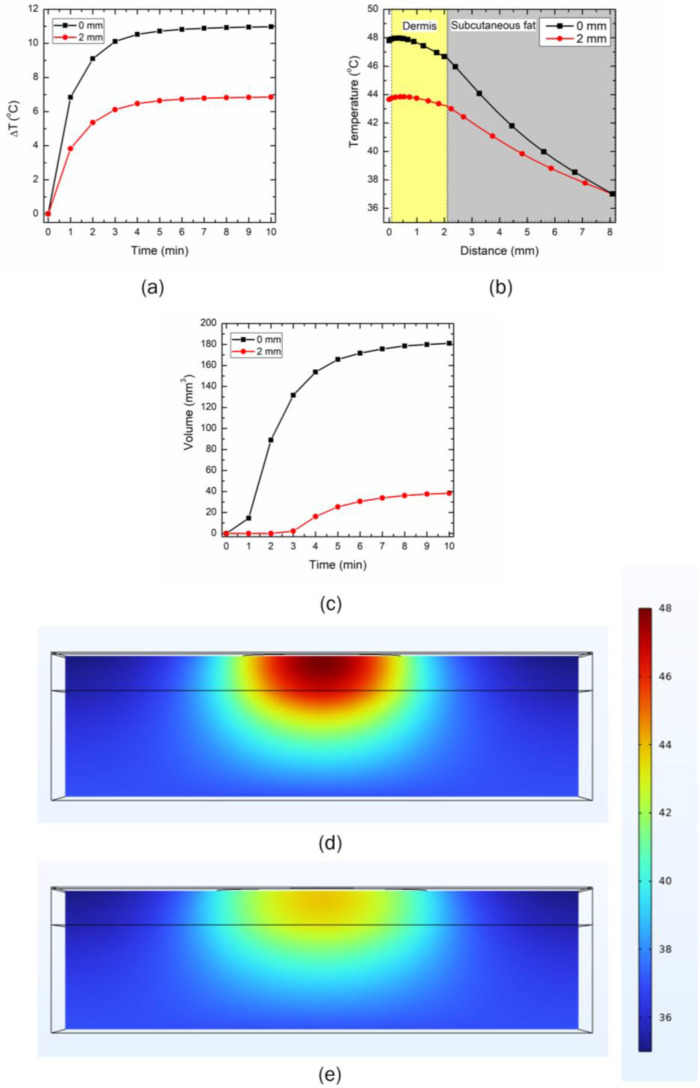
The effect of increasing the distance between the 90° LED (operating at 300 mW) and the skin surface on (**a**) the maximum temperature rise within the skin tissue, (**b**) the temperature distribution along the depth of the skin tissue, and (**c**) the heating volume attained with the T > 43 °C protocol. Temperature distribution (in °C) attained within the skin tissue after 10 min of LED irradiation, when the gap between the LED and skin surface was: (**d**) 0 µm, and (**e**) 2000 µm.

**Figure 10:**
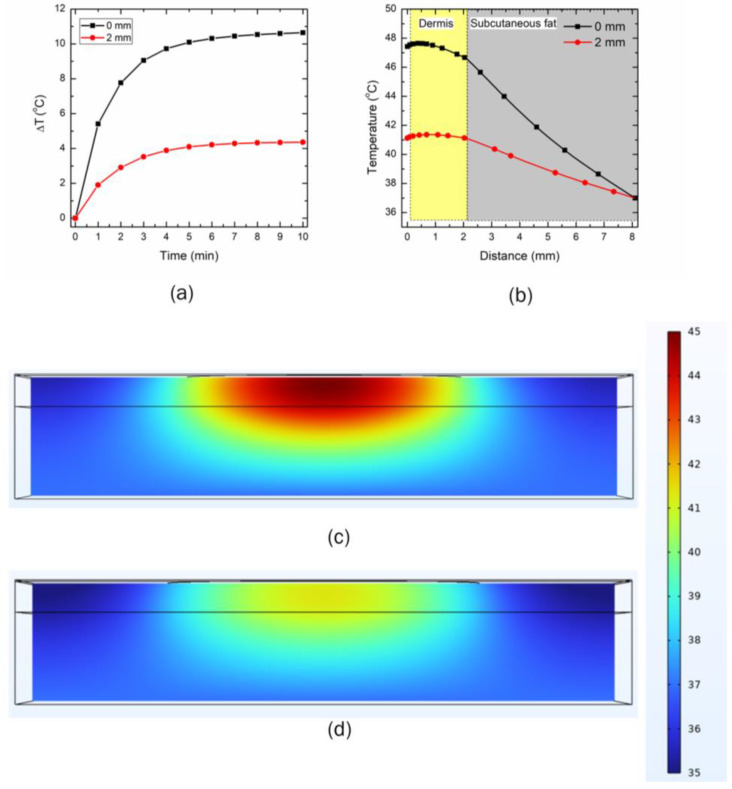
The effect of increasing the distance between the 150° LED (operating at 500 mW) and the skin surface on (**a**) the maximum temperature rise within the skin tissue, and (**b**) the temperature distribution along the depth of the skin tissue. Temperature distribution (in °C) attained within the skin tissue after 10 min of LED irradiation when the gap between the LED and skin surface is: (**c**) 0 µm, and (**d**) 2000 µm.

The effect of different distances between the LED and the skin’s topmost layer is presented in Figure 9 and Figure 10 for the 90° and 150° emission patterns, respectively. The LEDs were operated at the safe operating power of 300 mW and 500 mW for the 90° and 150° emissions, respectively. Two distances were selected for this analysis: (i) no gap between the LED and the skin (0 µm distance), and (ii) a gap of 2000 µm between the LED and the skin surface. Figure 9a presents the temporal variation in the maximum temperature for the two selected distances. As evident, a significant drop in the maximum temperature attained within the skin tissue prevailed, as the distance between the LED and the skin surface increased. After 10 min of LED therapy, the maximum temperature dropped by 37.6%, as the LED initially placed in direct contact with the skin surface was moved at a distance of 2000 µm from the skin surface. The temperature distribution attained along the depth of skin tissue after 10 min of therapy with the two selected locations for the 90° LED is presented in Figure 9b. The increase in the distance between the LED and the skin surface resulted in a drop in temperature at (a) the epidermis-dermis interface by 8.7%, and (b) the dermis-subcutaneous fat interface by 7.3%. This can be attributed to the fact that as the distance between the LED and the skin surface was increased, the LED irradiation intensity was no longer focused, and the losses were significant, thus resulting in an abrupt decline in the temperature attained within the skin tissue. For example, the standard deviation of the 90° LED beam Gaussian profile (σ) increased from 4.6 mm to 6.1 mm as the LED was moved at the distance of 2000 µm from the skin surface, thus leading to a decline in the irradiation intensity (as σ is inversely proportional to the irradiation intensity). The temporal variation in the volume obtained using the isotherm of 43 °C (i.e., the volume of skin tissue within the computational domain having temperature ≥ 43 °C) is shown in Figure 9c. As depicted in this figure, the temperature of 43 °C was attained almost instantaneously when the LED was placed in direct contact with the skin surface. In comparison, it took almost 3 min before the temperature of 43 °C was attained for the LED placed at a distance of 2000 µm from the skin surface. After 10 min of LED-based heating of the skin tissue, the desired heated volume of the tissue dropped abruptly by 372%, as the distance between the LED and the topmost layer of skin was increased to 2000 µm. Furthermore, the temperature distribution attained after 10 min of LED heating in the three-layered skin tissue is presented in Figure 9d and 9e, for 0 µm and 2000 µm, respectively.

Similar trends were obtained for the 150° LED, as presented in Figure 10. The maximum temperature dropped by 59% by increasing the distance between the LED and the skin to 2000 µm after 10 min of heating. Moreover, by increasing the distance, the temperature attained at (a) the epidermis-dermis interface dropped by 13.31%, and (b) the dermis-subcutaneous fat interface by 11.8%. Furthermore, the standard deviation of the 150° LED beam Gaussian profile (σ) increased from 7 mm to 10.7 mm as the LED was moved at the distance of 2000 µm from the skin surface, thereby leading to a decline in the irradiation intensity (Equation (3)). The temperature distribution attained within the skin tissue after 10 min of LED therapy is presented in Figure 10c,d. As evident from these figures, as the distance between the LED and skin increased to 2000 µm, the target temperature of 43 °C was not attained. Thus, the distance between the LED and the skin surface significantly affected the efficacy of the proposed LED-based therapy. This highlights that these distances can be altered to control the maximum temperature within the skin tissue, to provide patient-specific desired therapeutic effects.

### 3.4. Effect of Increase in the Number of LEDs on Temperature Rise within Skin Tissue

**Figure 11:**
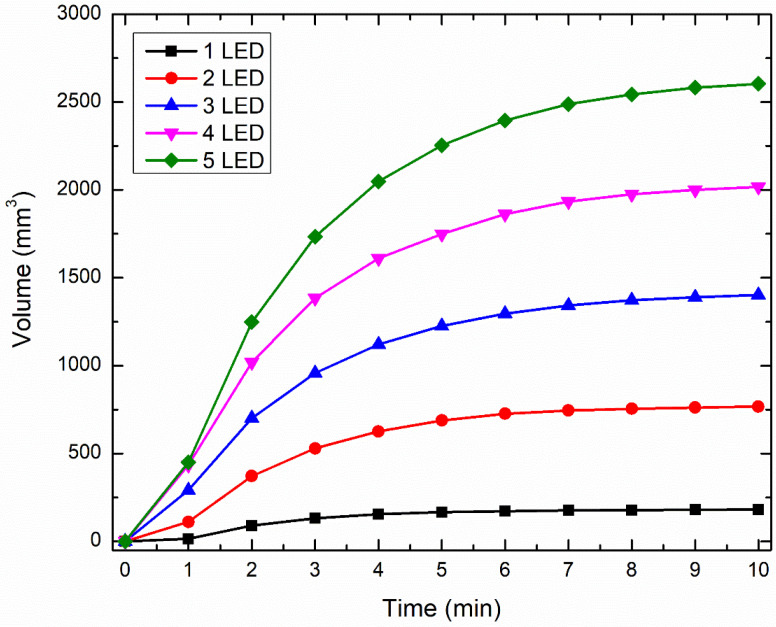
The effect of increased LEDs on the volume attained with the isotherm of 43 °C.

The analysis previously presented is made under the assumption that the precise location of the acupoint was previously known. Hence, the goal was to provide the thermal stimulus at the acupoint utilizing near-infrared LED that would further degranulate the highly concentrated mast cells. This would initiate biological mechanisms that would enhance vascular permeability and nerve activation, thereby providing therapeutic effects. The proposed LED-based therapy can also be extended to situations where the acupoint’s precise location is unknown. In such a scenario, the treatment goal would be to provide thermal stimulation to the large tissue area in contact with the LED, to achieve therapeutic effects. To attain this, a single LED would be insufficient, and multiple LEDs would have to be used and operated simultaneously, to heat up the large area/volume of the skin tissue. Thus, to include this in our numerical analysis, we present a case study highlighting the enhancement of the heated volume by using multiple LEDs operated simultaneously with the same power. We will proceed to present the results obtained with multiple 90° LEDs driven at the input power of 300 mW for 10 min. Figure 11 presents the temporal variation in the volume obtained using the isotherm of 43 °C, using 1, 2, 3, 4, and 5 LEDs. As evident from Figure 11, the increase in the number of LEDs leads to a substantial increase in the heated volume within the skin tissue. Compared to the single LED, the heated volume after 10 min of LED therapy increased by 324%, 674%, 1014%, and 1338% with the simultaneous usage of 2, 3, 4, and 5 LEDs, respectively. Moreover, the time required to attain the desired temperature of 43 °C also decreased with the increase in the number of LEDs, as shown in Figure 11.

**Figure 12:**
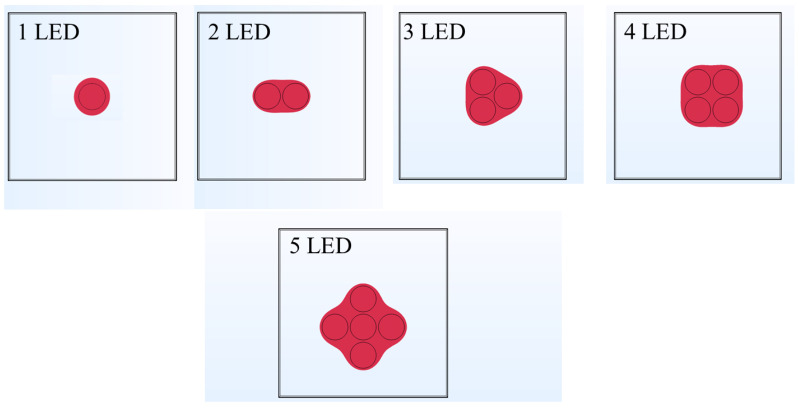
Pictorial representations of the top view of the isotherm of 43 °C on the skin surface attained with different numbers of LEDs.

A pictorial representation of the arrangement of these multiple LEDs is presented in Figure 12. Notably, the center-to-center distance of any LED from the one placed at the center was fixed at 10 mm for all configurations. Figure 12 also presents the heated volume as viewed from the top skin surface where the LED is applied, to give a pictorial representation of the volume enhancement with multiple LEDs. This analysis can be further extended to (a) optimize the distance between the LEDs, and (b) operating different LEDs at different powers, so as to restrict the maximum temperature attained within the skin tissue within safe limits, and provide the desired therapeutic effects.

## 4. Discussion

In this work, we proposed a novel acupuncture-like therapy, utilizing non-invasive LEDs to provide a physical stimulus to the skin tissue. The proposed therapy is completely non-invasive, eliminating the need to insert needles, as in conventional acupuncture therapy, or the production of smoke and odor from burning moxa sticks, as in conventional moxibustion. The application of visible blue and red LEDs is quite prevalent in several dermatological applications. In this work, we conducted a feasibility study to explore the effectiveness of the near-infrared LED to provide a thermal stimulus to the skin tissue. A computational framework was developed to quantify the spatiotemporal temperature distribution within the multilayer skin tissue irradiated by the near-infrared LED. Two commercially available LEDs with 90° and 150° FWHM angles operating at the wavelength of 850 nm were selected. Firstly, we experimentally characterized the irradiance profile of both these LEDs. A Gaussian profile was obtained for both LEDs, as presented in Figure 2. Secondly, we conducted the experimental tests utilizing both LEDs on the agar phantom gel, to obtain the temperature profile at a depth of 2 mm from the surface. The obtained profile was compared with the results predicted by the developed numerical model, incorporating the thermal and optical properties of the agar phantom under the same environmental conditions. A good agreement was obtained between the numerically predicted and experimentally obtained temperature profiles for both LEDs, as shown in Figure 4. Lastly, once the validation was complete, the numerical model was extended to incorporate multilayer skin tissue, utilizing the well-characterized properties for different layers of skin tissue available in the recent literature. The experimentally characterized Gaussian irradiance profile of the LED was applied at the top surface of the skin tissue to investigate the heat transfer attained within the skin tissue due to light-tissue interactions.

As this is a feasibility study, it is quite important to understand the influence of critical factors that could affect the efficacy of the proposed LED-based photothermal therapy. Thus, parametric studies were conducted to quantify the effects of the LED input power, treatment duration, and LED distance from the surface on the temperature distribution attained within the skin tissue for both the selected LEDs. The results of the parametric analysis of the above-mentioned critical factors are presented in Figure 5, Figure 6, Figure 7, Figure 8, Figure 9 and Figure 10. These results can be utilized to provide a safe optimal operating condition on the basis of the maximum temperature attained within the skin tissue. It is noteworthy to mention that the goal of the proposed therapy is to provide a thermal stimulus lower than the ablative temperature value of 50 °C, so as to minimize any irreversible damage to the skin tissue. Finally, this study also presented a case study related to the usage of multiple LEDs operating simultaneously, to enhance the volume/area of the thermal stimulus within the skin tissue, as shown in Figure 10 and Figure 11.

The main limitation of the developed model is related to the validation aspect. The developed numerical model was experimentally validated with the results obtained from the agar gel, the optical and thermal properties of which are inconsistent with skin tissue. Additionally, the validation was carried out in ex vivo settings, neglecting the critical impact of microvascular blood perfusion. Our group’s current research efforts are significantly devoted to addressing these limitations. Despite these limitations, we still believe that the results reported in this study will assist in better understanding the light-tissue interactions during LED therapy. Moreover, the parametric analysis results have highlighted several critical factors that can be targeted to enhance the efficacy of LED-based heating, to attain the desired therapeutic effects. For example, the linkage between the operating power of the LED and the maximum temperature attained within the skin tissue has been provided. Such information would be critical for designing an LED-based device capable of working under safe temperature limits. The results of the power-temperature relations presented in this study can be further utilized for developing different control algorithms to keep the maximum temperature within the skin tissue at safe operating conditions. These controllers (e.g., a real-time temperature feedback controller or power controller) can be designed by monitoring the temperature at the surface of the skin tissue, and modulating the applied power to keep the maximum temperature below the desired maximum value, to avoid any damage to the skin tissue, thus enhancing both the safety and efficacy of the proposed LED-based therapy. Our group is also working on the clinical translation of the reported numerical results in this work. We are working on developing a miniature device that could be easily mounted onto textile-based wearable products (e.g., a glove, head/arm/wrist/chest band, knee/neck pad, socks), and would be beneficial in providing on-demand physical stimulus to the wearer at home.

## 5. Conclusions

This study reports the development of a computational framework for accurately quantifying the heat transport within skin tissue exposed to near-infrared LED-based therapy. The numerical validation of the developed model has been carried out by performing experimental tests on agar phantom. A good agreement between the experimental and numerically predicted results was found. The numerical results evaluated the effect of the power, duration, distance between the LED and the skin, and number of LEDs on the maximum temperature attained within the skin tissue. It was found that the operating power and emission angle of the LED significantly affected the spatiotemporal temperature distribution obtained during LED-based therapy. Furthermore, the maximum temperature attained within the skin tissue could be controlled by altering the location between the skin surface and the LED. The application of multiple LEDs would result in an abrupt increase in the heated volume during LED-based photothermal heating, which could be optimized by using different distances between LEDs or operating LEDs at different power. It was found that the proposed LED-based therapy could safely provide thermal stimulus to the skin tissue, to a depth of 4–5 mm. This analysis will pave the way for quantifying the optimum settings for LED therapy, to operate within safe temperature limits. We expect that the future extension and clinical translation of this model will assist in designing a low-cost, miniature device capable of effectively and safely providing a thermal stimulus at home.

## Figures and Tables

**Table 1:** Thermal and optical properties of the various skin layers considered in the present study.

Parameters	Epidermis	Dermis	Subcutaneous Fat
Density, *ρ* (kg/m^3^)	1200	1090	1210
Specific heat capacity, *c_p_* (J/kg/K)	3950	3350	2240
Thermal conductivity, *k* (W/m·K)	0.24	0.42	0.194
Blood perfusion rate, *ω_b_* (1/s)	-	0.002	0.002
Absorption coefficient @ 850 nm, *µ_a_* (1/cm)	0.9	0.95	1
Reduced scattering coefficient @ 850 nm, *µ′_s_* (1/cm)	30	20	18

## Data Availability

All data generated or analyzed during this study are included in this article.
